# Supplementary figures and images for: Research on a rapid identification method for counting universal grain crops
Source: PLoS One. 2022 Sep 14;17(9):e0273785. doi: 10.1371/journal.pone.0273785 (PMC9473439; doi:10.1371/journal.pone.0273785)

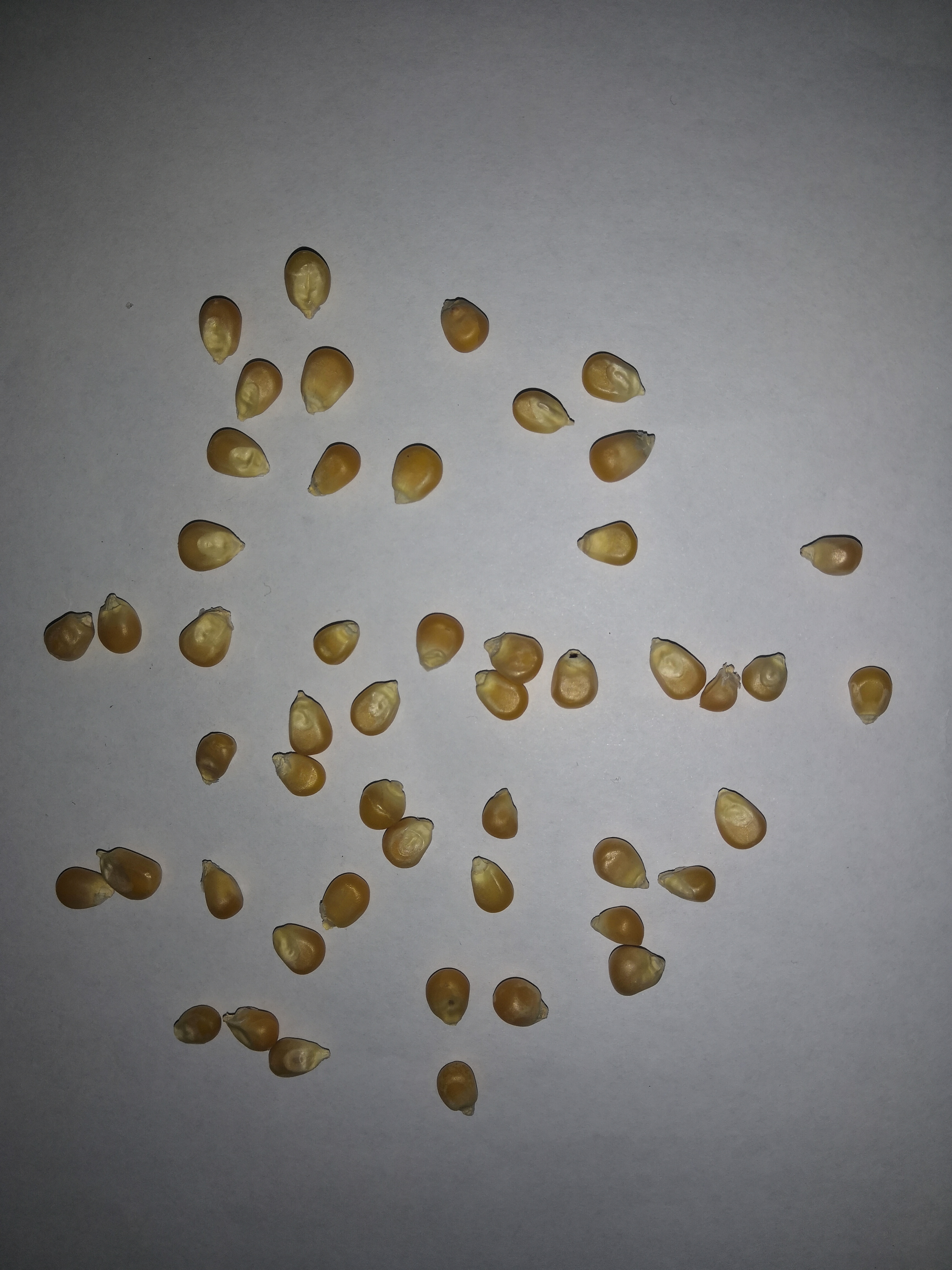

Supplement: S1 Fig — (JPG) [file pone.0273785.s001.jpg]
