# Supplementary material for: Research on a rapid identification method for counting universal grain crops
Source: PLoS One. 2022 Sep 14;17(9):e0273785. doi: 10.1371/journal.pone.0273785 (PMC9473439; doi:10.1371/journal.pone.0273785)
Supplement: Attachment — Submitted filename: Response to Reviewers.docx [file pone.0273785.s003.docx]

**Response to Reviewers**

ACADEMIC EDITOR:

The manuscript is dealing with very important subject that is extracting data using images. II have myself also read the manuscript and found this very useful for the scholars/students working in the area. The manuscript needs improvement by keeping in view comments of the reviewer. Some of my own comments are:

1. Improve presentation of data in tables and figures

Re: have modified Tables 1 - 5, Fig 2, Fig 7, Fig9, Fig11, Fig 12. The serial number has changed because Fig 10 has been removed.

2. The different steps involved may be presented in the form of flow chart

Re: Adjusted, demonstrated by flow chart.

3. Test files may be provided in the form of supplementary material

Re: The test images have been packaged and used as supplementary material, it contains test images of 6 different grains (144) and test results (excel). It is relatively large, with 460M of storage space.

4. Language and grammatical issues could be rectified.

Re: We have let an expert who is good at English to revise it. Please see the latest manuscript.

Reviewer #1:

This study proposes a grain counting algorithm with a wide range of applications, which supports the calculation of the number of grains on white paper by taking pictures of mobile phones. The method proposed in this paper has low cost, simple operation and strong applicability. The problems are as follows:

1.Special perspective analysis section, How to explain the missed detection and error of feature points in the sample diagram, it is recommended to expand the description.

Re: Modified as suggested. Added example descriptions and also labeled the images. The description is as follows.

As shown in Fig 7, where there was no Cpeak point above due to overlap, and only the Cpeak point below was detected, and calculation result of the corner point algorithm was 1.5, which was actually 2 grains.

2.The code of the volatility is inconsistent with the calculation formula in Evaluation index.

Re: Adjusted, the code should be S2, not S2.

3.In efficiency comparison section,there is no source for the counting machine with mechanical rotation takes 24 s.

Re: Adjusted, added citation to indicate the source of the data

4.The reference format of the two documents such as 31-32 is inconsistent with others.

Re: Adjusted, removed the citation article type

5.It is recommended to remove the content of the lightbox and focus on analyzing the white paper background.

Re: Adjusted, removed the contrast with the lightboxes as the background.

6.Capitalize the first letter in Figure S2.

Re: Adjusted, please see the updated picture.

7.In the evaluation of identification efficiency, "(3) The analysis time did not maintain a linear positive correlation with the number of grains in the picture. There were various factors that affected the running time, such as the tightness of the grains, size of the picture, and shape of the grains., etc." The conclusion of this sentence should be supported by corresponding experiments or cited.

Re: Adjusted, added citation support statement.

8.Pay attention to the expression of some phrases. Thousand grain, over segmentation, open source, short axis, etc. should have hyphens, "mega pixels" should be changed to "megapixels"; "light boxes" should be changed to "lightboxes".

Re: Adjusted as recommended.

9.Pay attention to plurals and grammar.such as“0.7 second”changed to“0.7 seconds”, "provide power support" changed to "provide powerful support" , "robust to variety changes" changed to "robust to various changes".

Re: Adjusted as recommended.

10.Pay attention to the expressions of some statements, such as "There were various factors that affected the running time" changed to "various factors affected……”, "as Lines …… show in Fig 4" changed to "as Lines …… shown in Fig 4".

Re: Adjusted as recommended.

11.The sentence is not clear: To improve the calculation efficiency and shorten the response time, the original image was specially compressed, but excessive compression affected the accuracy of the algorithm.

Re: Modified as suggested, as follows:

The resolution of the original photo was so large that uncompressed operations would greatly increase the algorithm processing time, but excessive compression would reduce the algorithm accuracy. To balance the accuracy and response time, the following conventional resolutions were tested: 1080, 1440, 1920, 2500, and 3000.
